# The STArgardt Remofuscin Treatment Trial (STARTT): design and baseline characteristics of enrolled Stargardt patients

**DOI:** 10.12688/openreseurope.13872.2

**Published:** 2022-07-22

**Authors:** Patty P.A. Dhooge, Philipp T. Möller, Camiel J.F. Boon, Andrew J. Lotery, Philipp Herrmann, Maurizio Battaglia Parodi, Wolfgang Klein, Mario G. Fsadni, Thomas H. Wheeler-Schilling, Oliver Jungmann, Hans Müller, Frank G. Holz, Steffen Schmitz-Valckenberg, Tobias M. Peters, Katarina Stingl, Carel B. Hoyng, Soraprazan Consortium

**Affiliations:** 1 Department of Ophthalmology, Radboud University Nijmegen Medical Centre, Nijmegen, 6500HB, The Netherlands; 2 Donders Institute for Brain, Cognition and Behaviour, Nijmegen, 6525EN, The Netherlands; 3 GRADE reading center, Bonn, 53127, Germany; 4 Department of Ophthalmology, University of Bonn, Bonn, 53127, Germany; 5 Department of Ophthalmology, Amsterdam University Medical Center, Amsterdam, 1081HV, The Netherlands; 6 Department of Ophthalmology, Leiden University Medical Center, Leiden, 2333ZA, The Netherlands; 7 Faculty of Medicine, University of Southampton, Southampton, SO17 1BJ, UK; 8 Center for Rare Diseases Bonn (ZSEB), University of Bonn, Bonn, 53113, Germany; 9 Department of Ophthalmology, Ospedale San Raffaele, Milan, 20132, Italy; 10 Katairo GmbH, Kusterdingen, 72127, Germany; 11 International Pharm-Med Ltd, Hemel Hempstead, HP1 1LD, UK; 12 University Eye Hospital, Center for Ophthalmology, Institute for Ophthalmic Research, University of Tübingen, Tübingen, 72076, Germany; 13 Smerud Medical Research Germany GmbH, Mannheim, D-68161, Germany; 14 John A. Moran Eye Center, University of Utah, Salt Lake City, Utah, 84132, USA; 15 Center for Rare Eye Diseases, University of Tübingen, Tübingen, 72076, Germany

**Keywords:** Stargardt disease, STGD1, ABCA4, Remofuscin, Soraprazan, qAF, quantitative autofluorescence

## Abstract

**Background: **This report describes the study design and baseline characteristics of patients with Stargardt disease (STGD1) enrolled in the STArgardt Remofuscin Treatment Trial (STARTT).

**Methods: **In total, 87 patients with genetically confirmed STGD1 were randomized in a double-masked, placebo-controlled proof of concept trial to evaluate the safety and efficacy of 20 milligram oral remofuscin for 24 months. The primary outcome measure is change in mean quantitative autofluorescence value of an 8-segment ring centred on the fovea (qAF _8_). Secondary efficacy variables are best corrected visual acuity (BCVA), low-luminance visual acuity (LLVA), mesopic microperimetry (mMP),  spectral domain optical coherence tomography (SD-OCT), reading speed on Radner reading charts, and patient-reported visual function as assessed by the National Eye Institute Visual Functioning Questionnaire 25 (NEI VFQ-25) and Functional Reading Independence (FRI) Index.

**Results: **Mean age of participants was 35±11 years with 49 (56%) female. Median qAF _8_ value was 438 Units (range 210-729). Median BCVA and LLVA in decimal units were 0.50 (range 0.13-0.80) and 0.20 (range 0.06-0.63), respectively. The median of the mean retinal sensitivity with mMP was 20.4 dB (range 0.0-28.8). SD-OCT showed median central subfield retinal thickness of 142 µm (range 72-265) and median macular volume of 1.65 mm ^3^ (range 1.13-2.19). Compared to persons without vision impairment, both reading performance and patient-reported visual function were significantly lower (p<0.001, one sample t-test). Mean reading speed was 108±39 words/minute with logRAD-score of 0.45±0.28. Mean VFQ-25 composite score was 72±13. Mean FRI Index score 2.8±0.6.

**Conclusions: **This trial design may serve as reference for future clinical trials as it explores the utility of qAF _8_ as primary outcome measure. The baseline data represent the largest, multi-national, STGD1 cohort to date that underwent standardized qAF imaging, reading speed assessment and vision-related quality of life measures which all contribute to the characterization of STGD1.

**EudraCT registration:** 2018-001496-20 (09/05/2019)

## Introduction

Stargardt disease (STGD1) is an inherited retinal disease caused by mutations in the *ABCA4* gene which results in progressive vision loss ^1, 2^. In the absence of a functional ABCA4 protein, bisretinoid products are formed and deposited in postmitotic retinal pigment epithelium (RPE) cells as lipofuscin ^3^. The lipofuscin build-up in the RPE of patients with STGD1 cannot yet be reversed and results in degeneration of RPE cells and, subsequently, corresponding photoreceptors leaving residual atrophy. This leads to worsening of visual acuity and could progress to blindness ^4^.

Currently, there is no treatment available which can remove the accumulated toxic lipofuscin to allow RPE cell regeneration. However, the ability of soraprazan to remove lipofuscin from RPE cells has been demonstrated in both primates and ABCA4-/- knock out mice ^5– 7^. Soraprazan was originally designed to treat gastro-oesophageal reflux disease (GERD). Its safety and tolerability following oral administration were already demonstrated in various phase I and phase II clinical trials ^8, 9^. To evaluate the safety and efficacy of oral soraprazan in patients with STGD1, we designed the Soraprazan Project as part of the European Union's Horizon 2020 research and innovation program (grant agreement No 779 317). Although the international non-proprietary name of the active ingredient remains soraprazan, the investigational drug is renamed remofuscin ^®^. The European Commission has granted an orphan designation (EU/3/13/1208) to use soraprazan, renamed remofuscin, for the treatment of STGD1. The STArgardt Remofuscin Treatment Trial (STARTT) is a phase II, prospective, multicentre, randomized, double-masked, placebo-controlled proof of concept trial. The current paper describes the study design of the STARTT and the baseline characteristics of enrolled patients.

The primary objective of the STARTT is to evaluate the efficacy of remofuscin in reducing the amount of lipofuscin in RPE cells of subjects with STGD1 by assessing the change in quantitative fundus autofluorescence (qAF) levels from baseline compared to placebo after treatment with remofuscin for up to 24 months. qAF acts as a marker of lipofuscin levels in the retina and allows for quantification of RPE changes at distinct retinal locations ^10^. With qAF being available since 2011, only limited data is available on qAF levels in STGD1 patients. In a single centre cohort of 77 STGD1 patients, 76,6% had higher qAF levels as compared to age-related controls ^11^. qAF levels were up to 8-fold higher in a different single centre STGD1 cohort of 42 patients ^12^. In addition, qAF was able to discriminate STGD1 from other retinal diseases that appear similar on clinical examination ^13– 15^. With 87 patients enrolled in the multicentre STARTT, this study represents the largest international STGD1 cohort to date that include repetitive, prospective standardized qAF imaging.

It is important to include outcomes parameters that are relevant from the patients’ perspective. Therefore, secondary efficacy parameters of the STARTT include best corrected visual acuity (BCVA), low luminance visual acuity (LLVA), mesopic microperimetry (mMP) and reading speed as functional measures. To our knowledge, Radner reading charts ^16^ have not been previously used to assess reading acuity and speed in patients with STGD1. In addition, we include patient-reported outcome measures regarding visual function and quality of life as assessed by the National Eye Institute Visual Functioning Questionnaire 25 (NEI VFQ-25) and Functional Reading Independence (FRI) Index ^17, 18^. Knowledge on the patient-reported vison-related quality of life of STGD1 patients is still limited. The baseline data presented in the current paper will therefore aid in characterizing the burden of STGD1 and provide outcome measures and possible indicators of cost effectiveness for all future clinical trials.

## Methods

STARTT is a randomized, double-masked, placebo-controlled study to evaluate safety and efficacy of oral remofuscin in subjects with STGD1 (EudraCT No. 2018-001496-20). An international consortium was formed consisting of six European investigator sites: two centres in the Netherlands (Radboud University Medical Centre Nijmegen, Nijmegen and Leiden University Medical Centre, Leiden), two centres in Germany (University Eye Hospital Tübingen, Tübingen and Department of Ophthalmology, University of Bonn, Bonn), one centre in the United Kingdom (University of Southampton, Southampton) and one centre in Italy (Ospedale San Raffaele, Milano), a Contract Research Organisation (CRO) (Smerud Medical Research), and a start-up company as a sponsor (Katairo). The study is conducted according to the principles of the Declaration of Helsinki (Version 2013) and is being managed and conducted according to the latest International Conference on Harmonisation (ICH) guidelines for Good Clinical Practice (GCP). For all sites, the study protocol was approved by the Institutional Review Boards (CCMO, project number NL68179.091.18; Commissie Mensgebonden Onderzoek Arnhem-Nijmegen, project number 2018-4988; BfArM, project number 4043121; Ethik-Kommission an der Medizinischen Fakultät der Eberhard-Karls-Universität and am Universitätsklinikum Tübingen, project number 843/2018AMG1; MHRA, project number 50612/001/001-0001; North-West Haydock Research Ethics Committee, project number 18/NW/0845; AIFA, project number 2018-001496-20; Comitato Etico Ospedale San Raffaele Milano, project number 2018-001496-20). The study was registered at www.clinicaltrialsregister.eu (EudraCT No. 2018-001496-20, registered May 9 ^th^, 2019). Potential subjects were identified and recruited from the investigator site’s database and all patients gave written informed consent before enrolment. Study staff were certified before patients were recruited. All study site personnel as well as the CRO personnel involved in the monitoring or conduct of the study were blinded to the individual subject treatment assignments. A data safety and monitoring board (DSMB) composed of a physician, an ophthalmologist not involved in the study conduct and a statistician reviews the blinded data on a regular basis in order to assure independent assessment and ensure safety of the subjects. Currently, the study is ongoing. Last patient last visit is planned for September 2022.

### Eligibility criteria

A complete list of inclusion and exclusion criteria is given in Table 1. The primary variable of interest is the change in mean qAF value of an 8-segment circular ring centred on the fovea (qAF _8_) from baseline to the key time point at Month 12 (Visit 10), and Month 24 (Visit 16). To be included, the qAF _8_ value must be ≥300 units at screening in the study eye. If the quality of the qAF image at screening is not deemed acceptable by the central reading centre, the qAF measurement is repeated at an additional visit (Visit 1B). Further, the study eye requires a BCVA between 0.20 and 0.80 (decimal units) at screening. If only one eye meets the above-mentioned criteria, this eye will be defined as the primary study eye. In case both eyes meet the criteria, the eye with the higher BCVA score will be defined as the primary study eye.

**Table 1.** Inclusion and exclusion criteria for STArgardt Remofuscin Treatment Trial.

	Criteria
Inclusion	– Male or female of any ethnicity and ≥ 18 years old – Clinical diagnosis of typical autosomal recessive Stargardt macular dystrophy (STGD1) – Genetic report indicating at least two *ABCA4* mutations (one with confirmed pathogenesis by a certified lab, one reported previously) – Onset of STGD1 disease before the age of 45 years – Elevated qAF _8_ in at least one eye at screening (value ≥ 300 units) – Visual acuity of the study eye: BCVA 0.2-0.8 (decimal unit) – Study eye must have clear ocular media and adequate pupillary dilation, including no allergy to dilating eye drops and with sufficient fixation to permit good quality retinal imaging. – Female subjects of childbearing potential and male subjects participating in the study who are sexually active must use acceptable contraception from screening and until one month after intake of the last IMP dose. Male and female subjects documented – as being of non-child bearing potential (e.g. infertile, surgically sterile, postmenopausal) are exempt from the contraceptive requirements. – Willing to avoid excessive exposure to sun light (e.g. by using a hat, ultraviolet absorbing sunglasses and sunscreen with a minimum SPF of 30)
Exclusion	– Intolerance to acid pump antagonists – Hypersensitivity to Soraprazan or to any of the excipients – Intake of prohibited medications/supplements (supplements containing vitamin A or beta-carotene, medications to treat any liver disease, or oral retinoid medications) within 28 days prior to screening and throughout the study – Intake of other drugs with a pH dependent absorption, e.g. ketoconazole – Breastfeeding or positive urine pregnancy test at screening or visit 2 (first intake of IMP). – At screening, clinically significant abnormal haematology or biochemistry findings or levels >1.5 x upper limit of normal of aspartate aminotransferase (AST), alanine aminotransferase (ALT), and/or total bilirubin – Acute or unstable severe disease or history of disease which in the opinion of the investigator would preclude participation in the study – Active or history of an additional ocular disorder in the primary study eye that, in the opinion of the investigator, may confound the study results. These include, but are not limited to, any reason that might interfere with the imaging techniques used in the study (such as optic media opacity or poor pupil dilatation), inflammatory eye – disease, other retinal disorders besides STGD, confirmed glaucoma or baseline intraocular pressure of ≥25mmHg, optic neuritis, high myopia (>8D spherical equivalent), amblyopia – Intraocular surgery or injections in the primary study eye within 180 days of the screening visit – Clinically significant abnormal electrocardiogram, or a corrected QT interval (QTc) of ≥450ms in males or ≥470ms in females – Participation in any other investigational clinical trial within 28 days of the screening visit

BCVA, best corrected visual acuity; IMP, investigational medicinal product; SPF, sun protection factor; STGD1, Stargardt disease; qAF _8,_ mean quantitative autofluorescence value of an 8-segment circular ring centred on the fovea

### Study treatment and randomization

In the STARTT, the investigational medicinal product (IMP) consists of two 10 milligram tablets, containing either remofuscin or placebo. Participants will have to take two IMP tablets orally per day in the late evening for up to 24 months. In the previous phase I and II trials using soraprazan to treat GERD, a daily dose of 20 milligram per day was considered safe ^8, 9^, and was used in STARTT. An oral dose of 6 mg/kg/day administered to monkeys for 1-year resulted in a full removal of lipofuscin without any ocular adverse effects. This dose in monkeys is approximately 3-fold higher than humans dosed at 20 mg/day. As a full effect on monkey lipofuscin removal was observed at 6 mg/kg/day after 12 months, it is assumed that a daily dose of 20 mg/day will also result in lipofuscin reduction in Stargardt disease patients. For placebo tablets, remofuscin is replaced by the same amount of cellulose. In order to minimize any symptoms related to the expected secondary pharmacodynamics effects on stomach acid reduction, dosing of remofuscin is planned before hours of sleep. The treatment is randomized in a 2:1 ratio (remofuscin:placebo).

### Visit procedures

The study consists of 17 standardized study visits spread over a review period of 24 months. Procedures took place within the investigator sites. Patients were screened between June 2019 and August 2020. The last patient visit is planned for September 2022. The study flow-chart is set out in Table 2 and Table 3. Comparison will be made between remofuscin and placebo treatment arms at month 12 (primary timepoint) and month 24 for the following functional and structural outcome measures: (1) qAF _8_ value; (2) BCVA taken in decimal as measured by Early Treatment Diabetic Retinopathy Study (ETDRS) charts; (3) BCVA taken in decimal under low luminance conditions by placing a 2.0-log-unit neutral density filter in front of the study eye and having the subject read a normally illuminated ETDRS chart (LLVA); (4) Low Luminance Deficit (LLD) calculated as the difference between BCVA and LLVA; (5) binocular and monocular maximum reading speed and critical print size as assessed by Radner reading charts ^16^; (6) macular functional response as assessed by mMP; (7) patient-reported visual function as assessed by the NEI VFQ-25 ^17^ and FRI Index ^18^; (8) central subfield retinal thickness (CSRT) and macular volume as assessed by SD-OCT; (9) macular atrophy as assessed by fundus autofluorescence (FAF); (10) changes on colour fundus photography (FP). In addition, the centre in Tübingen will assess changes in (11) pupillographic campimetry and (12) adaptive optics.

**Table 2.** Study flow-chart: screening to month 12.

Study Period	Screening		Randomisation	Treatment							
Clinic Visit (V) Number	V1	V1b (if required) ^6^	V2	V3	V4	V5	V6	V7	V8	V9	V10
Study Weeks (Months)	-4 to -1	between V1 and V2	0	4 (1)	8 (2)	12 (3)	16 (4)	24 (6)	32 (8)	40 (10)	52 (12)
Study Day	-28 to -7		0	28 (±3)	56 (±3)	84 (±3)	112 (±3)	168 (±3)	224 (±3)	280 (±3)	364 (±3)
Informed Consent	X										
Inclusion/Exclusion Criteria	X		X								
Review Medical history ^1^ (incl. Stargardt disease history) for any ongoing diseases	X										
Confirmation of ABCA4 mutations	X										
Check for concomitant medication	X		X	X	X	X	X	X	X	X	X
Bodyweight and Height ^2^	X										X
Physical examination	X										X
Vital signs	X		X	X	X	X	X	X	X	X	X
Eye examination	X		X		X		X	X	X	X	X
12-lead electrocardiogram	X		X	X	X	X	X	X	X	X	X
Blood sample for haematology and clinical chemistry ^3^	X		X	X	X	X	X	X	X	X	X
Urine pregnancy test (if applicable) ^4^	X		X	X	X	X	X	X	X	X	X
BEst corrected visual acuity (BCVA)	X		X	X	X	X		X		X	X
Low luminance visual acuity (LLVA), low luminance deficit (LLD)			X					X			X
Reading Speed			X								X
NEI VFQ25 and FRI Index			X					X			X
Retinal sensitivity (microperimetry)	BE ^5^		BE	BE	BE	BE		BE		BE	BE
Quantitative auto-fluorescence (qAF)	BE	BE	BE	BE	BE	BE	BE	BE	BE	BE	BE
SD-OCT	BE		BE	BE	BE	BE		BE		BE	BE
Colour Fundus Photography	BE		BE	BE	BE	BE		BE		BE	BE
Adaptive Optics ^7^			X					X			X
Pupillographic Campimetry ^7^			X					X			X
Definition of the “primary study eye”			X								
Adverse events	X		X	X	X	X	X	X	X	X	X
Randomisation			X								
IMP dispensation			X	X	X	X	X	X	X	X	
IMP return/accountability				X	X	X	X	X	X	X	X

^1^ongoing diseases or stopped within 12 months prior to screening

^2^height is only required at screening

^3^central laboratory

^4^urine stick test before IMP intake at the site

^5^BE = both eyes (examination of both eyes)

^6 ^V1b is required if the quality of the qAF image taken at V1 is not acceptable according to the central reading centre. In these cases, qAF should be repeated before V2

^7^only at the clinical trial site in Tübingen

**Table 3.** Study flow-chart: month 14 to month 24, including early termination (ET) visit and follow-up visit.

Study Period	Treatment						Follow up
Clinic Visit (V) Number	V11	V12	V13	V14	V15	V16 (or ET) ^1^	VS
Study Weeks (Months)	60 (14)	68 (16)	76 (18)	84 (20)	92 (22)	104 (24)	Last Visit (LV) +1
Study Day	420 (±3)	476 (±3)	532 (±3)	588 (±3)	644 (±3)	728 (±3)	LV +28 (±3)
Check for concomitant medication	X	X	X	X	X	X	X
Bodyweight							
Physical examination						X	
Vital signs	X	X	X	X	X	X	
Eye examination	X	X	X	X	X	X	
12-lead electrocardiogram	X	X	X	X	X	X	
Blood sample for haematology and clinical chemistry ^2^	X	X	X	X	X	X	X
Urine pregnancy test (if applicable) ^3^	X	X	X	X	X	X	X
BEst corrected visual acuity (BCVA)	X	X	X	X	X	X	X
Low luminance visual acuity (LLVA), low luminance deficit (LLD)						X	
Reading Speed						X	
NEI VFQ25 and FRI Index						X	
Retinal sensitivity (microperimetry)	BE ^4^	BE	BE	BE	BE	BE	BE
Quantitative auto-fluorescence (qAF)	BE	BE	BE	BE	BE	BE	BE
SD-OCT	BE	BE	BE	BE	BE	BE	BE
Colour Fundus Photography	BE	BE	BE	BE	BE	BE	BE
Adaptive Optics ^5^							
Pupillographic Campimetry ^5^							
Adverse events	X	X	X	X	X	X	X
IMP dispensation	X	X	X	X	X		
IMP return/accountability	X	X	X	X	X	X	

^1^ET = Early Termination, V16 will be performed

^2^central laboratory

^3^urine stick test before IMP intake at the site

^4^BE = both eyes (examination of both eyes)

^5^only at the clinical trial site in Tübingen

Safety values will include (1) evaluation of clinical and laboratory adverse events; (2) blood haematology and clinical chemistry; (3) vital signs including blood pressure and heart rate after at least 5 minutes of rest in supine; (4) 12 lead Electrocardiogram; (5) physical examinations; (6) body mass index (BMI); (7) number of patients with an increase in Lens Opacities Classification System grading by ≥2 grades.

### Retinal imaging

Retinal image acquisition and data transmitting are performed according to the standardized operating procedures from the Grading of Digital fundus Examination (GRADE) Reading Centre, Department of Ophthalmology, University of Bonn, Germany. This reading centre performs the standardized scientific analysis of the study endpoints involving qAF, FAF, mMP, SD-OCT, and FP. All equipment has been approved by the central reading centre and each photographer was certified prior to obtaining retinal images for the study.

***Microperimetry***. To assess retinal sensitivity and fixation, mMP is obtained using the CenterVue Macular Integrity Assessment (MAIA), Padova, Italy. With this device, fundus-controlled perimetry was performed with automated real-time fundus tracking. After at least 5 minutes of dark adaptation, retinal sensitivity was obtained with a custom-made test pattern using 54 Goldmann III stimuli of 200ms with an initial test brightness of 2.6±0.5 asb and a background luminance of 1.27cd/m2. The test pattern was automatically placed on the fovea and could be manually corrected by the instructor if needed. We use a custom-made test to ensure that microperimetry testing areas fall within the qAF grid as described by Delori. In this way we can correlate the qAF changes to the microperimetry changes in selected areas.

To reduce learning effects, a training exam containing 8 stimuli was conducted for both eyes before the custom-made test was performed and the study eye was always examined last. mMP always preceded qAF imaging, dilated ophthalmoscopy, and FP, as these procedures may temporarily affect retinal sensitivity. From the second visit onwards, the mesopic assessment of the screening visit serves as a reference and follow-up mode was used to automatically place all follow-up scans in the same location as the baseline scan. If Fixation Losses are above 30% or if the 95% Bivariate Contour Ellipse Area is above 50°, the test is deemed inaccurate and was repeated at least once. The mean MP sensitivity level was calculated per eye by averaging the point-wise retinal sensitivity in decibel.

***Quantitative fundus autofluorescence and fundus autofluorescence***. qAF and FAF images were obtained using a Spectralis device (Heidelberg Engineering, Heidelberg, Germany) with a qAF imaging mode including installation of the qAF reference standard and an upgrade to HEYEX software version 5.6 or higher. In order to reduce photopigment absorption, the retina was exposed to the blue excitation mode (488nm) for at least 20 seconds. For FAF, one image centred on the macula was obtained per eye (field of view 30° × 30°, 768 × 768 pixels, high-speed mode, 30 single frames using automated real-time (ART) modus). From the FAF images, if present, the macular atrophy, interpreted as complete loss of autofluorescence (analogous to definite decreased autofluorescence as described by Kuehlewein *et al*. ^19^), was calculated using the Heidelberg RegionFinder software version 2.6.4.0.

qAF imaging was performed according to the method developed by Delori *et al*. ^10^ Two qAF movies for both eyes were taken, each with a series of 12 frames per movie. Quality was evaluated so that every movie has at least 9 frames that are of equal brightness without shadowing or flickering. With the frames of good quality, a mean color-coded image was computed without normalization. The color-coded qAF images were adjusted for corneal curvature measurement performed at the screening visit and adjusted for patient’s age. The Delori pattern was placed, centred on the fovea and its border moved in direction of the optic nerve heads temporal border. Individual segmentation was performed to exclude atrophic areas and vessels and the mean grey level was calculated for each segment of the Delori pattern. qAF _8_ value was calculated by taking the mean of the grey levels in the 8 middle segments of the Delori pattern, as previously described by Delori *et al*. ^10^

***Spectral domain optical coherence tomography***. SD-OCT images were obtained with either the Spectralis OCT or Spectralis HRA+OCT device (Heidelberg Engineering, Heidelberg, Germany) using the Spectralis Software Version 6.9a or newer. Imaging consisted of different scan patterns per eye: (1) central line scan (30°, centred on the fovea, single B-scans, 30 frames for ART modus); (2) central volume scan (30° x 30°, centred on the fovea, 31 B-scans, 4 frames for ART modus); (3) enhanced-depth imaging (EDI) volume scan (30° x 25°, centred on the fovea, 241 B-scans, 9 frames for ART modus). The AutoRescan ^tm^ tool was used to automatically place all follow-up scans in the same location as the baseline scan. Central subfield retinal thickness (CSRT), and macular volume (centre 3 x 3 mm area) were calculated by manual segmentation.

***Fundus photography***. FP were acquired using a standard digital fundus camera (field 30–40°, minimum resolution of 1536 x 1536 pixels), centred on (1) optic disc; (2) fovea; (3) temporal part of macula.

### Sample size and baseline statistics

The sample size was calculated on the basis of the primary hypothesis that qAF _8_ score will be 50 units lower with active treatment compared to placebo after 12 months of treatment. Baseline qAF _8_ score was expected to be 484±109 units (mean± standard deviation (SD)) based on Burke *et al*. 2014 ^12^. With an assumed correlation of 0.75 between the qAF _8_ score values at baseline and after 12 months of treatment, the corresponding changes from baseline will have a standard deviation (SD) of 77 units. With 58 subjects on active treatment and 29 subjects on placebo, computer simulations show that analysis of covariance (ANCOVA) will have at least 80% power to detect a statistically significant treatment difference when using a 5% level of significance.

In this manuscript we report the descriptive statistics at baseline including mean ± SD, median, range and interquartile range for continuous variables and proportions for categorical variables. For Radner and NEI-VFQ 25, comparisons with previously published norm scores were assessed by a one sample t-test ^16, 17^. A p-value of <0.05 was considered significant. Statistical analysi *s* was performed using the SPSS statistics package for Windows; version 22 (SPSS IBM, New York, USA).

## Results

A total of 112 patients were screened of whom 87 patients fitted the inclusion criteria and were enrolled in the STARTT between June 2019 and September 2020 ^20^; 24 patients by the Radboud University Medical Centre Nijmegen, 12 patients by Leiden University Medical Centre, 23 patients by University Eye Hospital Tübingen, 8 patients by University of Bonn, 9 patients by University of Southampton and 11 patients by Ospedale San Raffaele. We included 49 females (56%) and 38 males (44%) with a mean age of 35 ± 11 years. Patients identified themselves with the following ethnicities: White (82 patients, 94%), Asian (3 patients, 4%), and Hispanic (2 patients, 2%). Age at onset of STGD1 was between 15 and 44 years with a median of 28 years.

The baseline study eye characteristics of all enrolled patients are summarized in Table 4. To provide a complete overview of qAF _8 _values, we used the screening values instead of the baseline values in 15 cases because of insufficient grading quality of the image (7 times), use of wrong settings (2 times) and incomplete data (6 times). At least one eye of all 87 enrolled patients had a qAF _8_ value ≥300 units and a BCVA between 0.20 and 0.80 (decimal units) at screening (visit 1). For 5 patients, qAF _8_ values for both eyes dropped below 300 units at the baseline visit (visit 2). Accordingly, baseline qAF _8_ values of the study eye were between 210 and 729 units with a median value of 438 units. For 4 patients, BCVA dropped below 0.20 at visit 2, resulting in baseline BCVA values between 0.13 and 0.80 with a median acuity of 0.50. If retinal imaging had poor quality, grading was not performed. Good quality mMP, FAF and SD-OCT images were available for 81, 83 and 82 eyes respectively. RPE atrophy was present in 42 of 83 gradable eyes (51%).

**Table 4.** Baseline study eye characteristics of patients enrolled in STArgardt Remofuscin Treatment Trial.

Study eye characteristics	Median (range, IQR)
**qAF _8_**	438 (210–729, 133)
**BCVA in decimals**	0.50 (0.13–0.80, 0.38)
**LLVA in decimals**	0.20 (0.06–0.63, 0.16)
**LLD in decimals**	0.18 (–0.05–0.56, 0.25)
**Mean retinal sensitivity in decibels (available for 81 eyes)** **Atrophic area in mm ^2^ (present in 42/83 gradable eyes)** **Central subfield retinal thickness in µm (available for 82 eyes)** **Macular Volume in mm ^3^ (available for 82 eyes)**	20.4 (0.0–28.8, 9.05) 0.97 (0.05–21.3, 4.58) 142 (72–265, 47.3) 1.65 (1.13–2.19, 0.28)

BCVA, best corrected visual acuity; IQR, interquartile range; LLVA, BCVA under low luminance conditions; LLD, Low Luminance Deficit; qAF _8_, mean quantitative autofluorescence value of an 8-segment circular ring centred on the fovea.

The patient-reported visual function was assessed with the NEI VFQ-25 in all 87 enrolled patients and the FRI in 86 patients, as 1 patient did not complete the FRI questionnaire. The results of the NEI VFQ-25 are reported in Table 5. Scores range from 0 to 100, where 100 indicates no disability ^17^. The overall NEI VFQ-25 composite score, averaging the vision-targeted subscale scores while excluding general health and the driving question, was 72±13. As compared to a reference group without eye diseases ^17^, STGD1 patients report a significantly lower visual function for almost all aspects (p<0.001, one sample t-test), except for general health (p=0.88, one sample t-test), colour vision (p=0.061, one sample t-test) and ocular pain (p=0.127, one sample t-test). The results of the FRI questionnaire are summarized in Table 6. FRI scores range from 1 to 4, with higher scores indicating greater independence. FRI level score is an ordinal classification using 4 functional levels: level 1, unable to do; level 2, help some or most of the time; level 3, moderately independent; level 4, totally independent ^18^.

**Table 5.** Results of the National Eye Institute Visual Function Questionnaire (NEI VFQ-25) at baseline.

Subscale	STGD1 patients (n=87), mean (SD)	Reference group without eye diseases ^1^, mean (SD)	Pairwise comparisons *, test value; p-value
General health	69 (20)	69 (24)	t=-0.148 ; p=0.88
General vision	58 (16)	83 (15)	t=-14.72; p<0.001
Near vision	59 (19)	92 (13)	t=-16.035; p<0.001
Distance vision	65 (18)	93 (11)	t=-14.549; p<0.001
Peripheral vision	81 (21)	97 (10)	t=-7.258; p<0.001
Colour vision	95 (14)	98 (8)	t=-1.897; p=0.061
Ocular pain	87 (16)	90 (15)	t=-1.541; p=0.127
Vision specific			
Role difficulties	63 (28)	93 (13)	t=-10.012; p<0.001
Dependency	75 (24)	99 (6)	t=-9.528; p<0.001
Social functioning	75 (20)	99 (3)	t=-10.924; p<0.001
Mental health	59 (21)	99 (3)	t=-18.048; p<0.001
Driving (n=43)	47 (16)	92 (12)	t=-18.171; p<0.001

SD, standard deviation; STGD, Stargardt disease.

*as assessed by one sample t-test

^1^Mangione CM, Lee PP, Gutierrez PR, Spritzer K, Berry S, Hays RD. Development of the 25-item National Eye Institute Visual Function Questionnaire. Arch Ophthalmol 2001;119(7):1050–8

**Table 6.** FRI index scores at baseline.

FRI Index	STGD1 patients (n=86)
FRI item-level score, mean (SD)	
Read written print such as books, magazines, newspapers	2.5 (1.0)
Read to pay bills or write a check	2.8 (0.9)
Read in order to take medicine	2.5 (1.1)
Read labels such as price tags, clothing labels, or food labels	2.7 (0.9)
Read numbers on a phone, answering machine, or caller-ID	3.3 (0.6)
Read words or numbers on screen while watching television	2.6 (1.1)
Read when using a computer	3.0 (0.6)
Overall FRI Index score, mean (SD) FRI Level, n (%)	2.8 (0.6)
FRI Level 1 (unable to do)	1 (1.2%)
FRI Level 2 (help some or most of the time)	19 (21.1%)
FRI Level 3 (moderately independent)	51 (59.3%)
FRI Level 4 (totally independent)	15 (17.4%)

One patient did not complete the FRI Index questionnaire. FRI, Functional Reading Independence; SD, standard deviation; STGD1, Stargardt disease. *one sample t-test, ^#^Overall Mann-Whitney U test

Besides the patient reported FRI, we measured reading speed directly using Radner reading charts. An overview of the results is presented in Table 7. Unfortunately, the reading task was not performed correctly in 20 patients, excluding these results from the analysis. As there are age-related changes in baseline reading acuity and speed, we compared our cohort to a reference group without eye diseases and with the same mean age of 35 years ^16^. Reading performance of our STGD1 cohort was significantly lower for all test values as compared to the reference group (p<0.001, one sample t-test).

**Table 7.** Results of Radner reading speed at baseline.

Binocular Radner Reading Chart Results	STGD1 patients (n=67), mean (SD	Reference group without eye diseases and age 35-39 ^1^, mean (SD)	Pairwise comparisons *, test value; p-value
Both eyes			
Maximum reading speed in words/minute	134 (50)	235.71 (24.48)	t=-16.682; p<0.001
Mean reading speed in words/minute	108 (39)	212.10 (21.92)	t=-21.750; p<0.001
Critical print size in logRAD	0.90 (0.24)	0.085 (0.0745)	t=28.321; p<0.001
Reading acuity in logRAD	0.46 (0.27)	-0.125 (0.044)	t=17.325; p<0.001
logRAD-score	0.45 (0.28)	-0.1020 (0.0436)	t=16.170; p<0.001

LogRAD, logarithm of the reading acuity determination (reading equivalent of logMAR); SD, standard deviation; STGD1, Stargardt disease. *as assessed by one sample t-test ^1^Radner W, Benesch T. Age-related changes in baseline reading acuity and speed as measured using RADNER Reading Charts in healthy eyes with best corrected ETDRS distance acuity. Br J Ophthalmol 2019;103(10):1518-23

## Discussion

The current paper describes the study design and baseline characteristics of the STARTT which evaluates the safety and efficacy of oral remofuscin in subjects with STGD1. In preclinical trials, remofuscin removed lipofuscin from RPE cells ^5– 7^. To measure the efficacy of remofuscin, qAF _8_, a direct marker of lipofuscin levels in the retina ^10^, was chosen as primary outcome measure for the STARTT. The STARTT study design is the first to explore the utility of qAF _8_ as clinical endpoint. qAF _8_ is considered to be a biomarker intended to detect both STGD1 activity and the pharmacologic activity of remofuscin.

qAF _8_ has high potential to become a valid and sensitive endpoint for clinical trials aiming to reduce or limit lipofuscin levels. Currently, atrophy area measured using FAF is the most widely used endpoint in phase II/III clinical trials for STGD1 ^21^. However, RPE atrophy progression over time is not adequate for use in patients with early disease stages, i.e. prior to the development of RPE atrophy, and assesses the disease when cell death is already present and cannot be prevented anymore. By contrast, qAF _8_ provides a quantifiable parameter for assessment of disease status, independent of disease stage, because qAF levels are elevated early in the disease course ^10, 12, 22^.

The use of qAF _8_ as primary efficacy endpoint in late-stage clinical trials is only acceptable if qAF _8_ is proven to reflect clinical benefit. According to the European Medicines Agency (EMA), a clinical endpoint should primarily evaluate how the patient functions or feels ^23^. The current trial design was therefore designed to be able to align qAF with functional endpoints including microperimetry, BCVA and reading speed. In addition, longitudinal qAF _8_ data from the STARTT will aid in further optimization of qAF _8_ technique and can be used to evaluate the intra-visit, inter-visit repeatability of qAF in a multi-center setting. In this way, the STARTT was uniquely designed to aid in the validation of qAF _8_ in order to make qAF _8_ acceptable to support regulatory decisions in the approval of a new drug ^24^. However, in order to truly establish the repeatability of qAF, a separate study should be set out.

For the STARTT, we specifically selected patients with preserved retinal function because they are likely to show disease progression without effective treatment. We therefore included patients with a BCVA between 0.20 and 0.80, because better baseline BCVA was associated with a greater yearly rate of decline ^25, 26^. The qAF inclusion criterium of 300 units was chosen on the basis of the natural disease course were qAF _8_ levels tend to initially increase, reach a ceiling level of approximately 800 units and then decline because of the development of RPE atrophy ^11, 12, 15^. The cut-off point of 300 units is well below the ceiling level, allowing us to measure progression, and above the average level of normal eyes ^11, 12^, allowing us to demonstrate a reduction of qAF _8_ levels if the treatment works. The highest measured qAF value at baseline was 729 units, thus none of the patients are close to the ceiling level. As atrophy was present in only 42 out of 83 gradable eyes (51%), half of the cohort has an early disease stage where qAF _8_ levels tend to increase. With these baseline values, we should be able to provide the first proof of effectiveness of remofuscin within 24 months if treatment works.

The STARTT represents the largest therapeutic trial to date to assess disease progression with functional measures, including reading speed assessment and patient-reported outcome measures (PRO’s) regarding visual function and quality of life. These data are of utmost importance to characterize the burden of STGD1 and provide outcome measures and possible indicators of cost effectiveness for all future clinical trials.

Both reading speed and VA are considered to be strong determinants of quality of life in STGD1 patients ^27^. Even though we included patients with preserved visual function, only 17.4% of patients claim to feel entirely independent as measured by the FRI index. However, a reference cohort without eye diseases does not exist yet for the FRI. The actual reading performance of our STGD1 cohort, as measured by Radner, was significantly lower for all test values as compared to a reference group ^16^ without eye diseases and with the same mean age of 35 years (p<0.001, one sample t-test).

With both reading speed and VA being lower than in a population without eye diseases, our patients report a considerable impact of STGD1 on daily function and quality of life as represented by the NEI VFQ-25. Even though general health was equally scored by STGD1 patients and the control group without eye diseases, STGD1 patients indicate significantly lower social functioning and mental health (p<0,001). This accords with earlier observations which showed that patients experience difficulties in the social environment (because of, among others, issues with facial recognition), tend to be frustrated and worried, have difficulties discussing their disease and even exhibit depressive symptoms ^28– 30^. A lower visual acuity, longer disease duration and younger age at onset resulted in a higher impact of STGD1 and a lower vision-specific quality of life ^28, 31^. The proposed treatment is targeted to remove lipofuscin which gives hope for recovery of RPE cell integrity, thereby restoring some visual function. Thus, if therapy proves to be effective, the quality of life of STGD1 patients might also improve. Because the STARTT includes PRO’s, we can calculate the quality-adjusted life years (QALYs) of individuals which is an important outcome measure that not only reflects clinical benefit, but also aids in assessing the value of medical interventions during economic evaluation ^32^.

In conclusion, the STARTT design demonstrates a unique approach to validate a new clinical endpoint (qAF _8_) while simultaneously evaluating the safety and efficacy of a new treatment agent for STGD1. The current trial design may therefore serve as reference for future clinical trials. For this, we reported a useful combination of multiple imaging methods, functional outcome measures, and PRO’s and described the qAF _8_ implementation in a multicentre setting. The results of reliability and repeatability of qAF _8_, the longitudinal progression of STGD1 including the qAF _8_ levels within this cohort and the safety and efficacy of oral remofuscin will be presented in subsequent publications. These publications will aid in detailed characterization about the natural course of STGD1, identify critical efficacy measures necessary to plan future clinical trials, and will of course demonstrate the safety and efficacy of oral remofuscin.

## Data availability

### Underlying data

DANS: The STArgardt Remofuscin Treatment Trial (STARTT): design and baseline characteristics of enrolled Stargardt patients. https://doi.org/10.17026/dans-x53-b696 ^20^.

This project contains the following underlying data:

- STARTT Baseline for repository.dat
- STARTT Baseline for repository.csv
- Codebook STARTT Baseline.pdf

Data are available under the terms of the Creative Commons Attribution 4.0 International license (CC-BY 4.0).
