# Supplementary material for: The STArgardt Remofuscin Treatment Trial (STARTT): design and baseline characteristics of enrolled Stargardt patients
Source: Open Res Eur. 2022 Sep 27;1:96. Originally published 2021 Aug 19. [Version 3] doi: 10.12688/openreseurope.13872.3 (PMC10445898; doi:10.12688/openreseurope.13872.3)
Supplement: Peer review history

# Peer review: Reviewer response for version 3

**DOI:** 10.21956/openreseurope.16362.r30158

**Published:** 2022-09-28

**Authors:** Victor Chong

**Affiliations:** 1 Janssen R&D LLC, Raritan, NJ, USA

No further comments.

Is the study design appropriate and does the work have academic merit?

Partly

Is the work clearly and accurately presented and does it cite the current literature?

Yes

If applicable, is the statistical analysis and its interpretation appropriate?

Not applicable

Are all the source data underlying the results available to ensure full reproducibility?

Partly

Are the conclusions drawn adequately supported by the results?

No

Are sufficient details of methods and analysis provided to allow replication by others?

Yes

Reviewer Expertise:

Clinical trial design and endpoint expert in retinal diseases

I confirm that I have read this submission and believe that I have an appropriate level of expertise to confirm that it is of an acceptable scientific standard.

# Peer review: Reviewer response for version 2

**DOI:** 10.21956/openreseurope.16203.r29743

**Published:** 2022-08-22

**Authors:** Victor Chong

**Affiliations:** 1 Janssen R&D LLC, Raritan, NJ, USA

I would like to thank the authors for answering my questions. It was very helpful. Most of the amendments clarify the reasoning and I hope that would strengthen the paper.

 My main reservation remains on qAF. I think we might be talking about something different here. Yes, anything can be used as a primary endpoint, but whether it would be a primary outcome measure for drug approval and whether this study would serve as a reference to achieve that position.

 My concerns as follows:

 A) qAF:

1. The variability is high in this study. In previously published work, they were mainly in single centre using single machine possibly by single operator. If the study is used as a reference, it would even weaken the case for qAF as the primary outcome measure, as the authors had noted qAF is more variable when the study becomes a multi-centre study as in this study. As they now have the baseline data, would the authors consider a recalculation of their study size? I am worried that the study is no longer power adequately. I understand that amendment at this stage is highly unlikely. But why this is not discussed as a limitation?
Functional correlation: The authors cited Muller *et al *2021. I quoted from the same paper as below.
  "When qAF, microperimetry, and ERG data were plotted together, a relationship unfolded in which high qAF was present in eyes with good retinal function. Although qAF was still in the upper range, retinal function deteriorated, and a significant reduction of qAF was consistently associated with loss of function" 
  Muller and colleagues found that high qAF were in eyes with good retinal function. A non-scientific interpretation of the data would be that high qAF is good for you. I do understand that high qAF is probably associated as earlier stage of the disease. As RPE cells die, the qAF reduces, and hence worse function. I understand that. As this study aims to reduce qAF, and that is the dissociation. Hence, my former comment on that there is no association of qAF with function. No suggestion that reduce qAF without RPE cell death is associated with visual function. In general, regulators would probably not accept that as approvable primary endpoint (outcome measure). This study might provide evidence that a reduction of qAF is related to better / improve visulal function as mentioned in the discussion. Nonetheless, as both qAF and visual function are highly variable. It might be hard to demonstrate that in such a small sample size. Might be worth discussing that as a limitation. Nonetheless, I am looking forward to the resutls.
  My suggestion is to weaken the wording of the conclusion. If anything, this trial might make qAF less likely as an approvable endpoint. I would not have thought that was the intention of the authors.

B) FAF:

 FAF lesion size was measured, and it is a potentially approvable endpoint. Why it is not included in the abstract and in the results as mentioned in my original comments. This looks odd? As a reviewer, something so obvious should be included and it is not included, I wondered why. In fact, with this sample size, it is more likely that a reduction of qAF is linked with slowing down of FAF lesion growth. FAF lesion size measurement is much less variable than visual function tests. FAF lesion growth is associated with long term visual loss. Why the omission? Why would the authors want to use something more variable (qAF) than something much easier (FAF)?

Is the study design appropriate and does the work have academic merit?

Partly

Is the work clearly and accurately presented and does it cite the current literature?

Yes

If applicable, is the statistical analysis and its interpretation appropriate?

Not applicable

Are all the source data underlying the results available to ensure full reproducibility?

Partly

Are the conclusions drawn adequately supported by the results?

No

Are sufficient details of methods and analysis provided to allow replication by others?

Yes

Reviewer Expertise:

Clinical trial design and endpoint expert in retinal diseases

I confirm that I have read this submission and believe that I have an appropriate level of expertise to confirm that it is of an acceptable scientific standard, however I have significant reservations, as outlined above.

# Response

**Published:** 2022-09-18

**Authors:** Patty Dhooge

**Affiliations:** 197667, The Netherlands

*Dear Dr. Chong, We understand your concerns and answer them below.*

 A) qAF: 1. The variability is high in this study. In previously published work, they were mainly in single centre using single machine possibly by single operator. If the study is used as a reference, it would even weaken the case for qAF as the primary outcome measure, as the authors had noted qAF is more variable when the study becomes a multi-centre study as in this study. As they now have the baseline data, would the authors consider a recalculation of their study size? I am worried that the study is no longer power adequately. I understand that amendment at this stage is highly unlikely. But why this is not discussed as a limitation?

***Answer:*** *We have changed our discussion section accordingly: The sample size was calculated based on the baseline qAF _8_ score of 484±109 units (mean± standard deviation (SD) by the single center, single operator study by Burke et al. 2014 ^12 ^. In our multi-center study, actual baseline values were slightly more variable, namely 438 (210–729, 133) median (range, IQR). We therefore chose to elongate the trial period from 12 to 24 months, Opposed to the cohort of Burke et al. 2014 ^12^, for the STARTT, we specifically selected patients with preserved retinal function because they are likely to show disease progression without effective treatment. We therefore included patients with a BCVA between 0.20 and 0.80, because better baseline BCVA was associated with a greater yearly rate of decline ^25, 26 ^. The qAF inclusion criterium of 300 units was chosen on the basis of the natural disease course were qAF _8_ levels tend to initially increase, reach a ceiling level of approximately 800 units and then decline because of the development of RPE atrophy ^11, 12, 15 ^. The cut-off point of 300 units is well below the ceiling level, allowing us to measure progression, and above the average level of normal eyes ^11, 12 ^, allowing us to demonstrate a reduction of qAF _8_ levels if the treatment works. The highest measured qAF value at baseline was 729 units, thus none of the patients are close to the ceiling level. As atrophy was present in only 42 out of 83 gradable eyes (51%), half of the cohort has an early disease stage where qAF _8_ levels tend to increase. With these baseline values, we should be able to provide the first proof of effectiveness of remofuscin within 24 months if treatment works.*  

 Functional correlation: The authors cited Muller *et al* 2021. I quoted from the same paper as below.   "When qAF, microperimetry, and ERG data were plotted together, a relationship unfolded in which high qAF was present in eyes with good retinal function. Although qAF was still in the upper range, retinal function deteriorated, and a significant reduction of qAF was consistently associated with loss of function"  Muller and colleagues found that high qAF were in eyes with good retinal function. A non-scientific interpretation of the data would be that high qAF is good for you. I do understand that high qAF is probably associated as earlier stage of the disease. As RPE cells die, the qAF reduces, and hence worse function. I understand that. As this study aims to reduce qAF, and that is the dissociation. Hence, my former comment on that there is no association of qAF with function. No suggestion that reduce qAF without RPE cell death is associated with visual function. In general, regulators would probably not accept that as approvable primary endpoint (outcome measure). This study might provide evidence that a reduction of qAF is related to better / improve visulal function as mentioned in the discussion. Nonetheless, as both qAF and visual function are highly variable. It might be hard to demonstrate that in such a small sample size. Might be worth discussing that as a limitation. Nonetheless, I am looking forward to the resutls.   My suggestion is to weaken the wording of the conclusion. If anything, this trial might make qAF less likely as an approvable endpoint. I would not have thought that was the intention of the authors.  

***Answer:*** *We have changed our discussion and conclusion section*: *The current trial design was therefore designed to be able to align qAF with functional endpoints including microperimetry, BCVA and reading speed. Nonetheless, as both qAF and visual function are highly variable. It might be hard to demonstrate a structure-function correlation in such a small sample size and future studies need to be undertaken.* *The wording of conclusion has been weakened: In conclusion, the STARTT design demonstrates a unique approach to explore the utility of qAF _8_ as primary outcome measure while simultaneously evaluating the safety and efficacy of a new treatment agent for STGD1.*    

 B) FAF:  FAF lesion size was measured, and it is a potentially approvable endpoint. Why it is not included in the abstract and in the results as mentioned in my original comments. This looks odd? As a reviewer, something so obvious should be included and it is not included, I wondered why. In fact, with this sample size, it is more likely that a reduction of qAF is linked with slowing down of FAF lesion growth. FAF lesion size measurement is much less variable than visual function tests. FAF lesion growth is associated with long term visual loss. Why the omission? Why would the authors want to use something more variable (qAF) than something much easier (FAF)?  

***Answer:*** *We did not include FAF lesion size, because most patients do not have definitely decreased fundus autofluorescence on FAF images at baseline as this was not part of the inclusion criteria. If atrophy (ddaf) is present, we will measure it, but we cannot track it in all patients and therefore, FAF lesion aria is not in the endpoints.*

# Peer review: Reviewer response for version 1

**DOI:** 10.21956/openreseurope.14956.r28352

**Published:** 2022-01-26

**Authors:** Victor Chong

**Affiliations:** 1 Janssen R&D LLC, Raritan, NJ, USA

The authors presented the STARTT trial design and reported the baseline characteristics. Overall, it was very well written. 

 My main concerns are as follows:

1. The main conclusion was that this trial design may serve as a reference for future clinical trials. I accept it MAY, but there is so far no evidence that qAF is correlated with visual function nor improvement of qAF is beneficial to the patient. In fact, qAF is not validated as a clinical endpoint in terms of reproducibility and clinical significance, either as a predictor of prognosis or visual function. In general, the primary endpoint of a trial should be a validated one. Hence, it is a bad practice. Nonetheless, I do agree that the information collected in this study might be able to validate qAF for future trials.
2. I am surprised that on one hand FAF was performed, but FAF area of atrophy changes were not included as an endpoint, as that is the usual acceptable clinical endpoint for Stargardt disease by regulatory agencies such as FDA. I understand there were patients included in the study with no atrophy at baseline. However, why the changes of the area of atrophy is not an endpoint?
3. Considering, 5 out of 87 patients (6%), the qAF dropped below the inclusion criteria of 300 units and based on the range, at least one patient dropped to 210 units. The hypothesis is that the reduction of qAF is 50 units. So at least with no treatment, at least one patient can drop close to 100 unit between screening and baseline. Furthermore, it was not reported how many other patients had changed qAF between screening and baseline. Once again highlighted the concern of using qAF as primary endpoint.
4. The choice of dosage is based on the study of the same drug in GERD. I assume that was failed. So is there any evidence other than it is safe that this dose has acceptable PK/PD to the retina / RPE. At least, some comments or some more information about that would be useful. The cited references (8 and 9), I was not able to access that to look for information on dose selection. For oral compounds, incorrect dosage is a common reason that a drug fails to show benefit, and since this dose is already failed in GERD, this should be looked into in more details. The authors might have done and it would be good if they can share that information in the article.

Minor suggestions: 

1. Letter counting is more commonly used for BCVA. And if decimal is used, it might be useful to know how part of the line read is included. Also it might be helpful to have Snellen equivalent as decimal BCVA is not commonly used in most parts of the world and often confused with LogMar BCVA.
2. Is there a particular reason to use a 54 custom-made test pattern for Stargardt? Had that been validated?

Is the study design appropriate and does the work have academic merit?

Partly

Is the work clearly and accurately presented and does it cite the current literature?

Yes

If applicable, is the statistical analysis and its interpretation appropriate?

Not applicable

Are all the source data underlying the results available to ensure full reproducibility?

Partly

Are the conclusions drawn adequately supported by the results?

No

Are sufficient details of methods and analysis provided to allow replication by others?

Yes

Reviewer Expertise:

Clinical trial design and endpoint expert in retinal diseases

I confirm that I have read this submission and believe that I have an appropriate level of expertise to confirm that it is of an acceptable scientific standard, however I have significant reservations, as outlined above.

# Response

**Published:** 2022-07-19

**Authors:** Patty Dhooge

**Affiliations:** 197667, The Netherlands

Dear dr. Chong, Thank you for reviewing our manuscript entitled “The STArgardt Remofuscin Treatment Trial (STARTT): design and baseline characteristics of enrolled Stargardt patients” We feel strengthened by the positive comments and useful suggestions we have received. Please find enclosed our detailed point-by-point response to your review.  

- ***The main conclusion was that this trial design may serve as a reference for future clinical trials. I accept it MAY, but there is so far no evidence that qAF is correlated with visual function nor improvement of qAF is beneficial to the patient. In fact, qAF is not validated as a clinical endpoint in terms of reproducibility and clinical significance, either as a predictor of prognosis or visual function. In general, the primary endpoint of a trial should be a validated one. Hence, it is a bad practice. Nonetheless, I do agree that the information collected in this study might be able to validate qAF for future trials.***

The EMA has confirmed during protocol advice in 2014 (EMEA/H/SA/2776/1/2014/PA/SME/III), that qAF analysis can be accepted as a primary endpoint in clinical studies. This has also been confirmed at a meeting with the Medicinal Product Agency, Sweden. Multiple studies report repeatability coefficients of 6-12% for intra-visit repeatability 7-14% for inter-visit repeatability (interval up to 64 days).(Delori, Greenberg et al. 2011, Greenberg, Duncker et al. 2013, Burke, Duncker et al. 2014, Duncker, Greenberg et al. 2014, Duncker, Tsang et al. 2015, Duncker, Tsang et al. 2015, Muller, Gliem et al. 2015, Reiter, Told et al. 2019)  Measures of qAF were correlated with retinal function assessed by full-field electroretinography (ERG) and fundus-controlled perimetry.(Müller, Gliem et al. 2021) 

 We believe that the use of qAF as endpoint in this proof-of-concept study qAF is justified because of its special property that it is an early, direct, and objective marker of lipofuscin levels in the retina. Of course, we will also assess the secondary endpoints in order to analyze the efficacy of Remofuscin.  

- ***I am surprised that on one hand FAF was performed, but FAF area of atrophy changes were not included as an endpoint, as that is the usual acceptable clinical endpoint for Stargardt disease by regulatory agencies such as FDA. I understand there were patients included in the study with no atrophy at baseline. However, why the changes of the area of atrophy is not an endpoint?***

Macular atrophy as assessed by fundus autofluorescence (FAF) was included as clinical trial endpoint number 9. See paragraph ‘visit procedures’ and ‘retinal imaging’: “From the FAF images, if present, the macular atrophy, interpreted as complete loss of autofluorescence (analogous to definite decreased autofluorescence as described by Kuehlewein et al. 19 ), was calculated using the Heidelberg RegionFinder software version 2.6.4.0.”  

- ***Considering, 5 out of 87 patients (6%), the qAF dropped below the inclusion criteria of 300 units and based on the range, at least one patient dropped to 210 units. The hypothesis is that the reduction of qAF is 50 units. So at least with no treatment, at least one patient can drop close to 100 unit between screening and baseline. Furthermore, it was not reported how many other patients had changed qAF between screening and baseline. Once again highlighted the concern of using qAF as primary endpoint.***

Indeed, the real-life test-retest variability of qAF seems higher than assumed. We will take this increased variability into account when analyzing the data.  

- ***The choice of dosage is based on the study of the same drug in GERD. I assume that was failed. So is there any evidence other than it is safe that this dose has acceptable PK/PD to the retina / RPE. At least, some comments or some more information about that would be useful. The cited references (8 and 9), I was not able to access that to look for information on dose selection. For oral compounds, incorrect dosage is a common reason that a drug fails to show benefit, and since this dose is already failed in GERD, this should be looked into in more details. The authors might have done and it would be good if they can share that information in the article.***

An oral dose of 6 mg/kg/day administered to monkeys for 1-year resulted in a full removal of lipofuscin without any ocular adverse effects. This dose in monkeys is approximately 3-fold higher than humans dosed at 20 mg/day. As a full effect on monkey lipofuscin removal was observed at 6 mg/kg/day after 12 months, it is assumed that a daily dose of 20 mg/day will also result in lipofuscin reduction in Stargardt disease patients. We will add this information to our manuscript.  

**Minor suggestions: **

- ***Letter counting is more commonly used for BCVA. And if decimal is used, it might be useful to know how part of the line read is included. Also it might be helpful to have Snellen equivalent as decimal BCVA is not commonly used in most parts of the world and often confused with LogMar BCVA.***

We will add Snellen equivalents to the manuscript.
 

1. ***Is there a particular reason to use a 54 custom-made test pattern for Stargardt? Had that been validated?***

We use a custom-made test to ensure that microperimetry testing areas fall within the grid as described by Delori. In this way, we can correlate the qAF changes to the microperimetry changes in selected areas. We will add this information to the manuscript.

# Peer review: Reviewer response for version 1

**DOI:** 10.21956/openreseurope.14956.r28043

**Published:** 2021-12-15

**Authors:** Xiangrong Kong

**Affiliations:** 1 School of Medicine, Johns Hopkins University, Baltimore, MD, USA

This manuscript reports the study design and baseline characteristics of the STARTT study, a randomized placebo controlled parallel clinical trial on Stargardt disease. There were 6 clinical sites from Europe. Participants were randomized to the intervention 20mg Soraprazan per day or placebo with a ratio of 2:1, and are followed monthly for 24 months. Strengths of the study and manuscript include the high significance given the unmet need for Stargardt disease management, pre-clinical studies supporting the trial to test repurposing of Soraprazan to Stargardt disease, the known safety profile and potential low cost of Soraprazan, many outcome modalities including 2 instruments measuring patient reported outcomes and multiple visual function outcomes such as low illuminance acuity and reading speed, and the clear description of the study design in the manuscript. The primary outcome measure was based on the change of qAF level between baseline and 24 months. This is novel and compared to the FAF atrophy area, qAF can be used for earlier stage patients where sizable FAF lesions have yet to form. In addition to evaluating the safety and efficacy of the drug, the longitudinal data collected through the trial will provide natural history information on disease progression measures such as qAF, low luminance VA, reading speed and patient reported outcomes, which in and of itself will contribute to the better understanding of the disease progression as there is very limited literature currently on the longitudinal changes of these measures for Stargardt.

 A few comments are listed below.

- Page 4, DSMB preforms reviews of the blinded data regularly. There are two considerations here, first, if the DSMB are all masked from the treatment information in the data, there is the question of how to timely identify safety signals. The drug’s safety profile is relatively well known from other conditions, but this trial requires long-term use of the drug. Long-term safety needs to be tracked timely during the trial. It is unclear how effective safety tracking can be done if the DSMB is masked. Second, considering the randomization ratio of 2:1, not equal allocation, it is easy to surmise the group with larger sample size would be the intervention. More details would be helpful in the manuscript regarding how the data are presented to DSMB if they need to be blinded.
- Page 5 about eligibility criteria and study eye selection were described clearly. One question is why only use one study eye from a patient. The intervention tested is a systemic treatment. Also as an inherited disease, it is most often bilateral presentation and it seems the study is measuring both eyes on most imaging/tests. Using data from both eyes if both are eligible will provide more information and will be more statistically powerful. It is unclear why the study design would only consider one study eye for primary efficacy analysis.
- The study flow-chart is very nicely presented. Participants are followed monthly for 2 years. Such follow-up frequency is intensive for patients especially these are patients with visual impairment. Most of the testing/imaging are also done monthly. This can be burdensome for patients. For FAF imaging, there is the theoretical concern that the intense short-wavelength (SW) light excitation during imaging may be detrimental to Stargardt eyes (Cideciyan A.V., *et al.* 2007 *Opt Soc Am A Opt Image Sci Vis.*) and reduced illuminance SW imaging protocol has been used in Stargardt imaging. Was this considered in the imaging protocol for this trial? It would be helpful to mention the justification of monthly visits and imaging. Also, patients of Stargardt must be highly motivated, but missed visits can occur especially with such a frequent schedule. Are there measures to help prevent missed visits?
- The baseline data presented are very informative. They suggest the Radner reading test is not a good test for Stargardt patients, as a nontrivial proportion of participants could not preform it correctly. The comparison of NEI-VFQ response to a reference group is interesting and well thought of. The significantly lower scores on all scales except general health and ocular pain are informative and expected.
- Discussion Page 12 first paragraph on the left, “longitudinal qAF _8_ data from the STARTT will aid in further optimization of qAF _8_ technique and can be used to test the reliability and repeatability of qAF _8. _” . Two comments can be made here. First, normally when designing a trial, the primary outcome measure chosen should be a measure that’s already been shown to be able to be measured reliably. A reliable outcome measure can minimize bias and increase power for detecting treatment efficacy. Second, assessments of the reliability and repeatability of qAF measure require its own study, including repeated gradings of the same qAF image for image grading repeatability and multiple images for the same eye within a short period for imaging technique reliability. The longitudinal data as currently described in the manuscript cannot be used for assessing reliability and repeatability of the measure as there would be true biological change in qAF that is confounded with the measurement’s own variability due to image grading and imaging process.
- Discussion Page 12 second paragraph on the left about the natural disease trajectory of qAF is very interesting and informative. So the qAF change in Stargardt is not monotone over time. With the trial sample including half participants who already had FAF atrophy at enrollment, where their qAF changes are expected to fall on the natural disease trajectory during the 2 years of follow-up? If their qAF increase to the plateau and then decrease during the 2 years, how to use the value of the change of qAF between baseline and 24 months visits to infer disease course during the 2 years?
- Discussion Page 12 first paragraph on the right, regarding “Thus, if therapy proves to be effective and visual acuity and reading ability improve, the quality of life of the STGD1 patients would also improve”, there are two comments. First, this suggests that the proposed treatment is targeted to improve visual function. It is questionable whether such restoring visual function target can be achieved with the presented mechanism of how Soraprazan may work in Stargardt (removing lipofuscin in RPE). It’s worth to note that the therapeutic target of delaying disease progression is not the same as the target of reversing visual function loss or improving visual function. If Soraprazan indeed can remove lipofuscin in RPE, it may delay disease progression, but it is unclear how it may reverse the RPE and photoreceptor loss that has already occurred prior to treatment initiation. Second, the baseline quality of life (QOL) of Stargardt patients is worse compared to normal controls. But to evaluate whether a treatment can improve QoL in the patients, it is important to know whether and how QoL as measured by NEI-VFQ changes over a 2-years period.

Is the study design appropriate and does the work have academic merit?

Yes

Is the work clearly and accurately presented and does it cite the current literature?

Yes

If applicable, is the statistical analysis and its interpretation appropriate?

Yes

Are all the source data underlying the results available to ensure full reproducibility?

Yes

Are the conclusions drawn adequately supported by the results?

Partly

Are sufficient details of methods and analysis provided to allow replication by others?

Yes

Reviewer Expertise:

Clinical trial design, statistics, Stargardt disease natural history

I confirm that I have read this submission and believe that I have an appropriate level of expertise to confirm that it is of an acceptable scientific standard.

## References

[1] : Reduced-illuminance autofluorescence imaging in ABCA4-associated retinal degenerations. *J Opt Soc Am A Opt Image Sci Vis* .2007;24(5) : 10.1364/josaa.24.001457 1457-67 10.1364/josaa.24.001457 17429493PMC2579898

# Response

**Published:** 2022-07-19

**Authors:** Patty Dhooge

**Affiliations:** 197667, The Netherlands

Dear dr. Kong,   Thank you for reviewing our manuscript entitled “The STArgardt Remofuscin Treatment Trial (STARTT): design and baseline characteristics of enrolled Stargardt patients” We feel strengthened by the positive comments and useful suggestions we have received. Please find enclosed our detailed point-by-point response to your review.

- ***Page 4, DSMB performs reviews of the blinded data regularly. There are two considerations here, first, if the DSMB are all masked from the treatment information in the data, there is the question of how to timely identify safety signals. The drug’s safety profile is relatively well known from other conditions, but this trial requires long-term use of the drug. Long-term safety needs to be tracked timely during the trial. It is unclear how effective safety tracking can be done if the DSMB is masked. Second, considering the randomization ratio of 2:1, not equal allocation, it is easy to surmise the group with larger sample size would be the intervention. More details would be helpful in the manuscript regarding how the data are presented to DSMB if they need to be blinded.***

The DSMB receives a sub-set of data relevant for safety assessment. The assessment may include but is not limited to: adverse events, safety laboratory tests (haematology and clinical chemistry), qAF8 level, Lens Opacities Classification System grade. The DSMB is blinded to individual subject treatment assignments for the duration of the entire study, but may be un-blinded in order to facilitate prompt analysis of any safety/tolerance issues that may arise. Also, pharmacovigilance staff may un-blind single cases in relation to SUSAR reporting.
** **

- ***Page 5 about eligibility criteria and study eye selection were described clearly. One question is why only use one study eye from a patient. The intervention tested is a systemic treatment. Also as an inherited disease, it is most often bilateral presentation and it seems the study is measuring both eyes on most imaging/tests. Using data from both eyes if both are eligible will provide more information and will be more statistically powerful. It is unclear why the study design would only consider one study eye for primary efficacy analysis.***

Initially, we will look at data from one study eye as standard statistical methods can be employed when analyses are based on a single eye per individual. If needed, second eye data can be included to confirm results.  

- **The study flow-chart is very nicely presented. Participants are followed monthly for 2 years. Such follow-up frequency is intensive for patients especially these are patients with visual impairment. Most of the testing/imaging are also done monthly. This can be burdensome for patients. For FAF imaging, there is the theoretical concern that the intense short-wavelength (SW) light excitation during imaging may be detrimental to Stargardt eyes (Cideciyan A.V., et al. 2007  Opt Soc Am A Opt Image Sci Vis.) and reduced illuminance SW imaging protocol has been used in Stargardt imaging. Was this considered in the imaging protocol for this trial? It would be helpful to mention the justification of monthly visits and imaging. Also, patients of Stargardt must be highly motivated, but missed visits can occur especially with such a frequent schedule. Are there measures to help prevent missed visits?**

Indeed, reduced illuminance SW imaging would be an option if the primary goal is to slow down the progression of RPE atrophy as reduced illuminance SW imaging can well be used to measure areas of definitely decreased autofluorescence (i.e. RPE atrophy). However, STARTT focusses on hyper autofluorescent signals, since the mechanism of action of Remofuscin concerns the removal of lipofuscin. Due to its autofluorescence, the amount of lipofuscin in the RPE cells – and therefore also any signs of removal – can be directly analyzed by quantitative autofluorescence (qAF). qAF is not possible with reduced illuminance SW-AF imaging. Patients are therefore already exposed to more intense light during SW-AF imaging. Making additional FAF images requires minimal extra time. Besides, the maximal retinal irradiance during SW-AF and qAF imaging is approximately 2 mW/cm2 for a 10 degrees by 10 degrees image and is therefore well below international standards.(Bindewald, Schmitz-Valckenberg et al. 2005) However, those thresholds are mainly based on unaffected retinas, while diseased retinas may have lower light-damage thresholds. Indeed, simulations suggest that the RPE of STGD1 patients is generally at increased risk of photo-oxidative stress and that exposure during FAF imaging will amplify this risk.(Teussink, Lambertus et al. 2017) However, this theoretical phototoxicity has never been verified with in-vivo data.   Currently, participating in a clinical trial is the only option for STGD1 patients to receive treatment. Therefore, patients are highly motivated. In order to prevent missed visits, planning of visits is discussed and coordinated together with patients, and we offer patients reimbursement of all costs.
** **

- ***The baseline data presented are very informative. They suggest the Radner reading test is not a good test for Stargardt patients, as a nontrivial proportion of participants could not preform it correctly. The comparison of NEI-VFQ response to a reference group is interesting and well thought of. The significantly lower scores on all scales except general health and ocular pain are informative and expected.***

Thank you for your compliment.

- ***Discussion Page 12 first paragraph on the left, “longitudinal qAF8 data from the STARTT will aid in further optimization of qAF8 technique and can be used to test the reliability and repeatability of qAF8. ” . Two comments can be made here. First, normally when designing a trial, the primary outcome measure chosen should be a measure that’s already been shown to be able to be measured reliably. A reliable outcome measure can minimize bias and increase power for detecting treatment efficacy. Second, assessments of the reliability and repeatability of qAF measure require its own study, including repeated gradings of the same qAF image for image grading repeatability and multiple images for the same eye within a short period for imaging technique reliability. The longitudinal data as currently described in the manuscript cannot be used for assessing reliability and repeatability of the measure as there would be true biological change in qAF that is confounded with the measurement’s own variability due to image grading and imaging process.***

The EMA has confirmed during protocol advice in 2014 (EMEA/H/SA/2776/1/2014/PA/SME/III), that qAF analysis can be accepted as a primary endpoint in clinical studies. This has also been confirmed at a meeting with the Medicinal Product Agency, Sweden. Indeed, reliability of qAF has not yet been established in STGD1. Multiple studies report repeatability coefficients of 6-12% for intra-visit repeatability 7-14% for inter-visit repeatability (interval up to 64 days).(Delori, Greenberg et al. 2011, Greenberg, Duncker et al. 2013, Burke, Duncker et al. 2014, Duncker, Greenberg et al. 2014, Duncker, Tsang et al. 2015, Duncker, Tsang et al. 2015, Muller, Gliem et al. 2015, Reiter, Told et al. 2019)  Indeed, to establish repeatability of qAF, a separate study should be set out. However, before the initiation of treatment, all patients satisfying the other enrollment criteria of this trial underwent qAF imaging at two visits, with two qAF imaging sets per visit. The interval between both visits was between 6-72 days likely providing a small biological change in qAF during this period. Thus, by using these individual measurements, we are able to evaluate the intra-visit, inter-visit repeatability of qAF in a multi-center setting. We agree that the longitudinal data as currently described in the manuscript cannot be used for assessing reliability and repeatability and we will correct the wording.

- ***Discussion Page 12 second paragraph on the left about the natural disease trajectory of qAF is very interesting and informative. So the qAF change in Stargardt is not monotone over time. With the trial sample including half participants who already had FAF atrophy at enrollment, where their qAF changes are expected to fall on the natural disease trajectory during the 2 years of follow-up? If their qAF increase to the plateau and then decrease during the 2 years, how to use the value of the change of qAF between baseline and 24 months visits to infer disease course during the 2 years?***

Before analysis of the qAF level, atrophic areas were excluded by an individual segmentation based on the qAF add-on tool in the HEYEX software (Heidelberg Engineering, Heidelberg, Germany), and with manual alterations if deemed necessary. We believe that the cut-off point of 300 units is well below the ceiling level, allowing us to measure progression, and allowing us to demonstrate a reduction of qAF _8_ levels if the treatment works. In addition, we can use the microperimetry analysis to differentiate. If treatment works, retinal sensitivity will remain the same or improve when qAF levels decrease. If treatment does not work, retinal sensitivity will decline when qAF levels decline.
** **

- ***Discussion Page 12 first paragraph on the right, regarding “Thus, if therapy proves to be effective and visual acuity and reading ability improve, the quality of life of the STGD1 patients would also improve”, there are two comments. First, this suggests that the proposed treatment is targeted to improve visual function. It is questionable whether such restoring visual function target can be achieved with the presented mechanism of how Soraprazan may work in Stargardt (removing lipofuscin in RPE). It’s worth to note that the therapeutic target of delaying disease progression is not the same as the target of reversing visual function loss or improving visual function. If Soraprazan indeed can remove lipofuscin in RPE, it may delay disease progression, but it is unclear how it may reverse the RPE and photoreceptor loss that has already occurred prior to treatment initiation. Second, the baseline quality of life (QOL) of Stargardt patients is worse compared to normal controls. But to evaluate whether a treatment can improve QoL in the patients, it is important to know whether and how QoL as measured by NEI-VFQ changes over a 2-years period.***

Indeed, proposed treatment is targeted to remove lipofuscin which gives hope for recovery of RPE cell integrity, thereby restoring some visual function. However, RPE and photoreceptor loss cannot be reversed.  We will change the wording. To the best of our knowledge, studies on NEI-VFQ changes over a 2-year period in STGD1 or IRDs have not been carried out so far. STARTT is a placebo-controlled study. Therefore we can compare NEI-VFQ changes between both arms in order to evaluate if treatment can improve the quality of life in STGD1 patients.
